# Deoxyschizandrin Loaded Liposomes on the Suppression Lipid Accumulation in 3T3-L1 Adipocytes

**DOI:** 10.3390/molecules23092158

**Published:** 2018-08-27

**Authors:** Xiaona Liu, Shifeng Wang, Zhisheng Wu, Zhaoyi Wang, Qiusheng Zheng, Defang Li

**Affiliations:** 1 School of Integrated Traditional Chinese and Western Medicine, Binzhou Medical University, Yantai 264003, China; xiaonaliu5627@163.com (X.L.); zqsyt@sohu.com (Q.Z.); 2 School of Chinese Materia Medica, Beijing University of Chinese Medicine, Beijing 100102, China; wanshifen@aliyun.com (S.W.); zywang6834@126.com (Z.W.)

**Keywords:** deoxyschizandrin, nonalcoholic fatty liver disease, liposomes, lipid droplet, 3T3-L1 adipocyte

## Abstract

Deoxyschizandrin (DS) is a bioactive benzocyclooctadiene lignan found in the fruit of *Schisandra chinensis*. However, poor bioavailability and non-specificity of DS frequently caused low therapeutic efficacy. In the present study, DS-liposome (DS-lipo) was implemented to enhance the hepatic targeting and inhibition effects on adipocyte differentiation in 3T3-L1 cells. The formulations enabled encapsulation of as much as 24.14% DS. The DS-lipo prepared was about 73.08 nm, as measured by laser light scattering (LLS) morphology. In the visual field of a scanning electron microscope (SEM), the liposomes were spherical with similar size and uniform dispersion. Fluorescence live imaging study exhibited hepatic targeting of liposomes in vivo. Furthermore, High-Content Analysis (HCS) imaging microassay analyses revealed DS-lipo and DS reduced cytoplasmic lipid droplet in 3T3-L1 adipocytes, with the IC_50_ value of 8.68 μM and 31.08 μM, respectively. The lipid droplet accumulation inhibition rate of 10 μM DS-lipo was above 90%, which was even superior to the effect of 30 μM DS solution. The current findings suggest that DS-lipo was a therapeutic strategy for alleviating lipid-associated diseases and nonalcoholic fatty liver disease (NAFLD).

## 1. Introduction

Lipid droplets are intracellular organelles that play a vital role in cellular lipid storage and trafficking. Overloaded intracellular lipid droplets can intimately contribute to metabolic diseases. Nonalcoholic fatty liver disease (NAFLD) is a chronic liver disorder, which is characterized by excessive lipid accumulation in the liver. The incidence of NAFLD is increasing with high-energy diets and fast human lifestyles, with the risk even extending to children [1,2]. Therefore, the development of agents that can alleviate lipid droplet accumulation could be a therapeutic strategy for NAFLD.

*Schisandra chinensis* (Turz Baill) (*S. chinensis*) (SC) is an essential herb in Traditional Chinese Medicine (TCM). It was recorded in the “Shen Nong’s herbal classic” thousands of years ago and included in the Chinese Pharmacopoeia [3]. *Schisandra chinensis* has been traditionally used in China in the treatment of liver damage and poor liver function by facilitating detoxifying activity [4,5]. SC was evaluated for inhibition effects on adipocyte differentiation. A previous study reported that SC inhibited adipocyte differentiation and lipid accumulation in 3T3-L1 preadipocytes [6]. The extract of SC was reported to ameliorate NAFLD [7]. Particularly, deoxyschizandrin (DS), the major bioactive lignin of *Schisandra chinensis*, exhibited beneficial biological activities, that is, a hepatoprotective effect, and antitumor and antioxidant actions [8,9,10]. However, the low aqueous solubility of DS restricts its clinical applicability. Therefore, an idea for the preparation of a DS delivery vehicle is highly desired to improve biodistribution in vivo.

Liposomes attribute features are biocompatibility, biodegradability, low toxicity, and targeting properties which offer novel pharmaceutical formulations for those drugs which cannot be administered by conventional treatments [11,12]. Given their amphiphilic nature, the nanocarriers can improve solubility of active compounds [13,14]. Moreover, the primary role of drug carriers is to deliver therapeutic components to the diseased tissue and to increase tissue selectivity in vivo. Research has demonstrated that the accumulation of nanoparticles in different organs mainly depends on their size. Especially, nanospheres (~100 nm) were preferentially distributed in liver [15]. Thus, liposome delivery systems were implemented to enhance molecular transport and pharmacological effectiveness. Our work assessed the liver-targeting and the potency of DS-lipo on lipid metabolism. To monitor the microdistribution of nanocarriers in vivo, Fluorescence live imaging (FLI) was implemented in this study. We used 1,1′-dioctadecyl-3,3,3′,3′-tetramethylindocarbocyanine iodide (DiR) [16,17,18], a near-infrared fluorescent dye, for liposome tracing in vivo imaging. Furthermore, we used 3T3-L1 adipocytes, a good model for adipogenesis investigation in vitro, to study the effect of DS-lipo on inhibiting adipogenesis.

## 2. Results and Discussions

### 2.1. Liposome Characterization

Quantitative analysis of the DS drug was studied. The linearity range of the calibration curves was y = 59.363x + 1.0455 in the range of 5.0–51.5 μg/mL with a correlation coefficient of 0.9999 and y = 66.295x + 3.8003 in the range of 0.214–2.57 μg/mL with a correlation coefficient of 0.9994. The LOD and LOQ of DS were 0.049 and 0.149 mg/mL, respectively. The values obtained for encapsulation efficiency and drug loading of DS were 24.14 ± 0.19% and 1.34 ± 0.01%, respectively.

**Figure 1:**
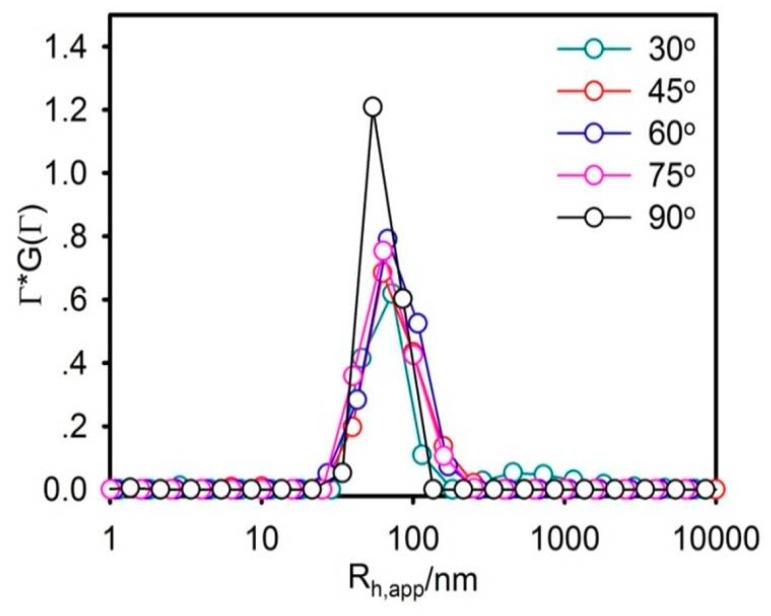
Size distribution of deoxyschizandrin and its liposome (DS-lipo). ρp/l = 1.0. Temperature = 37 °C.

**Figure 2:**
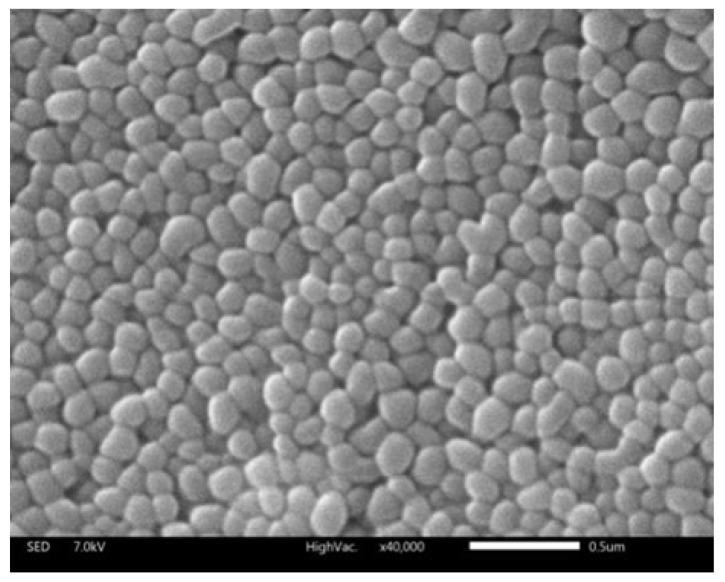
SEM image of DS-lipo. Notes: Magnification: ×50,000. Scale bar = 0.5 μm.

The particle size should be rigidly controlled due to the injection administration. Moreover, it is one of the most vital parameters that determine the in vitro and in vivo outcomes of the nanocarrier. In this work, the average particle size of liposomes was about 73.08 nm and perfectly exhibited a narrow size distribution, using DLS, as shown in Figure 1. The morphology of the liposome was observed by SEM (Figure 2). The liposomes were spherical in SEM images with a uniform size of below 100 nm, which was relatively consistent with DLS observations.

Previous research revealed that aggregation in the liver is obvious when the particle size is less than 100 nm [19,20]. It is trapped by the sinusoidal tubules of liver and spleen to reach the passive targeting role. The obtained size range and morphology suggested that liposomes were small unilamellar vesicles (SUVs) and suitable for further study [21]. The zeta potential of liposomes was found to be slightly positive, with zeta potentials of 1.3 mV.

### 2.2. Evaluation of the DS-Lipo In Vitro

#### 2.2.1. Stability of DS-Lipo in Serum

**Figure 3:**
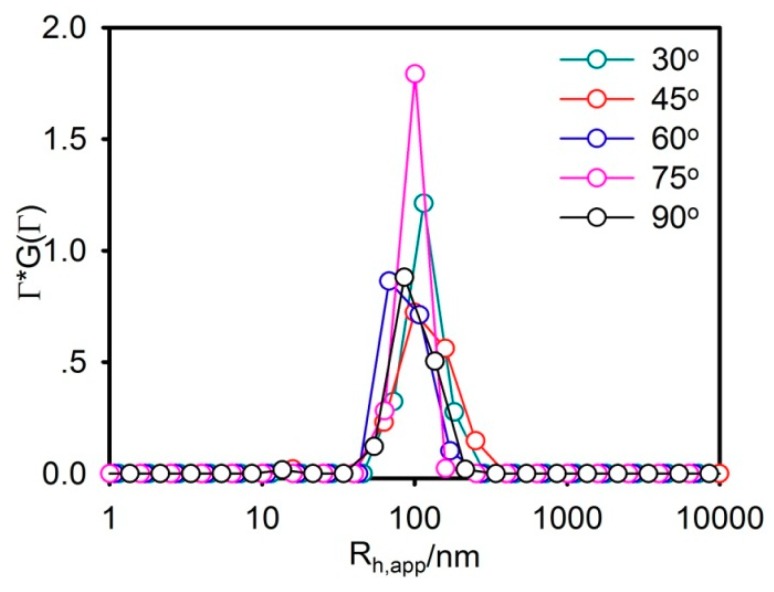
Size distribution of DS-lipo in serum at 24 h. ρp/l = 1.0. Temperature = 37 °C.

A 50% FBS solution at pH 7.4 was used to simulate the physiological conditions in the stability of DS-lipo in serum. In serum at pH 7.4, the average particle size of liposomes was about 88.6 nm, as shown in Figure 3. No significant size changes were observed in the average size and distribution of DS-lipo over 24 h, which indicated that there was no aggregation of liposomes in the presence of serum. It meant that there was no aggregation of DS-lipo in the presence of serum over 24 h.

#### 2.2.2. In Vitro Release Study 

**Figure 4:**
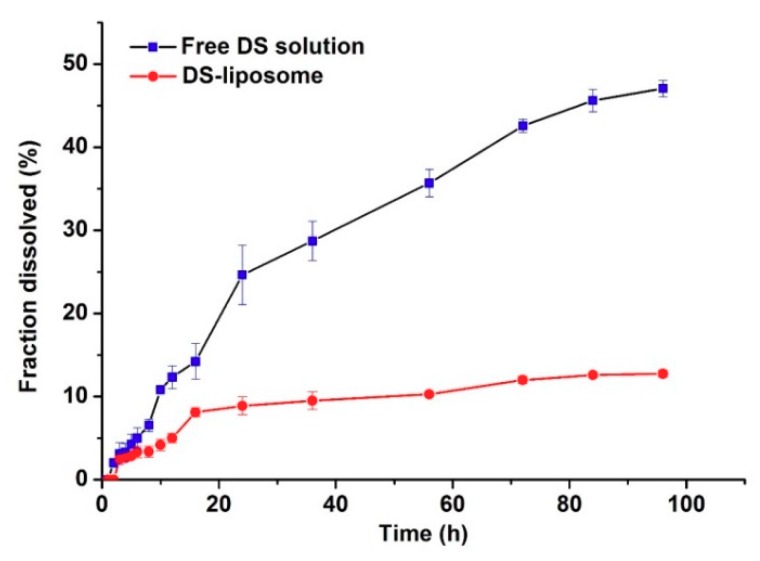
In vitro release of drugs from free DS solution and DS-lipo.

Figure 4 exhibits the in vitro release profiles of DS-lipo and free DS solution. The concentration of DS was 0.04 mg/mL equivalent to liposomes formulation in vitro release study. Notably, the release rate of DS-lipo was significantly lower than that of free drug and the liposomal-based formulation showed a prominent sustained profile. Free DS showed an apparently monophasic release with uniform speed throughout the study: approximately 16% of the free drug was released in the initial 24 h, then another 38% of the drug was further released during the subsequent 72-h incubation. However, only 13% of the total drug was released of DS-lipo within 96 h of incubation, with 5% of the entrapped drug in the initial 8 h, followed by an additional 8% release over the next 88 h. This sustained phenomenon may account for the retention effect of liposomes. No initial burst of drug release appeared in either group. Subsequently, the in vitro release data of liposomal formulation were kinetically analyzed, which was fit to Higuchi’s model with relatively high-correlation coefficient values.

### 2.3. Increased Liver Targeting of Liposome In Vivo

**Figure 5:**
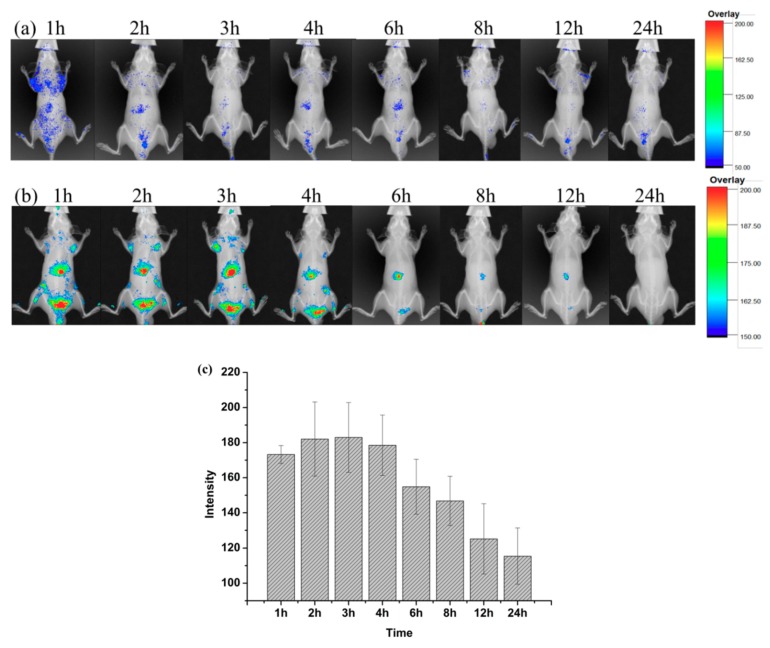
The fluorescent image in vivo and vitro (**a**), (**b**) free 1, 1′-dioctadecyl-3,3,3′,3′-tetramethylindocarbocyanine iodide (DiR)-solution and DiR-liposome in vivo NIR fluorescence imaging at the designated times post injection, (**c**) The fluorescent intensity at different time points corresponding with (**b**), (Values represent mean ± SD of 3 animals.).

Figure 5a,b. shows the time-dependent in vivo fluorescence imaging of DiR-liposome and free DiR-solution (as the control) in male BALB/c-nu mice. It was found that the two groups released completely different uptake patterns. Free DiR molecules resulted in a poor fluorescent signal that was hardly observed when the background of control groups was adjusted to the same level (Figure 5a). In contrast, the notable fluorescence was preferentially accumulated in the liver region of rats, as shown in Figure 5b. It ceased slowly and was still detectable at 12 h post-injection of DiR-labeled nanoparticles. The results illustrated the hepatic targeting of liposomes. The tissue distribution was measured by performing the living-body imaging with DiR. As shown in Figure 5c, semiquantitative analysis of the in vivo fluorescent images in the liver revealed that more DiR-liposome was taken up by the liver from 1 to 3 h. The fluorescent signal was the strongest at 3 h. Subsequently, the fluorescent intensity gradually decreased, then disappeared completely at 24 h. However, the fluorescence of the free DiR-solution group in the liver region was relatively weak at both time points. The above results elucidated that liposomes exhibited a dominant uptake in the liver.

The distinct fates between free DiR and DiR-loaded liposomes may be attributed to their distinct biodistribution mechanisms in vivo. Free DiR is a low molecular weight substance that can be distributed systemically in the body and transported across cellular membranes by passive diffusion, which is what resulted in its low uptake in the liver, as expected, owing to the lipophilic nature and low concentration gradient. Small particle sizes of liposomes (below 100 nm) have also contributed to long-term retention in the liver [22]. Poste also reported that ultrastructural and cell-fractionation studies revealed more efficient penetration of smaller unilamellar vesicles across liver sinusoids than the larger multilamellar vesicles [23]. Therefore, nanoparticles showed an extended circulation profile with better liver accumulation than free DiR.

### 2.4. Effect of DS-Lipo on Lipid Droplet Accumulation in 3T3-L1 Adipocytes

**Figure 6:**
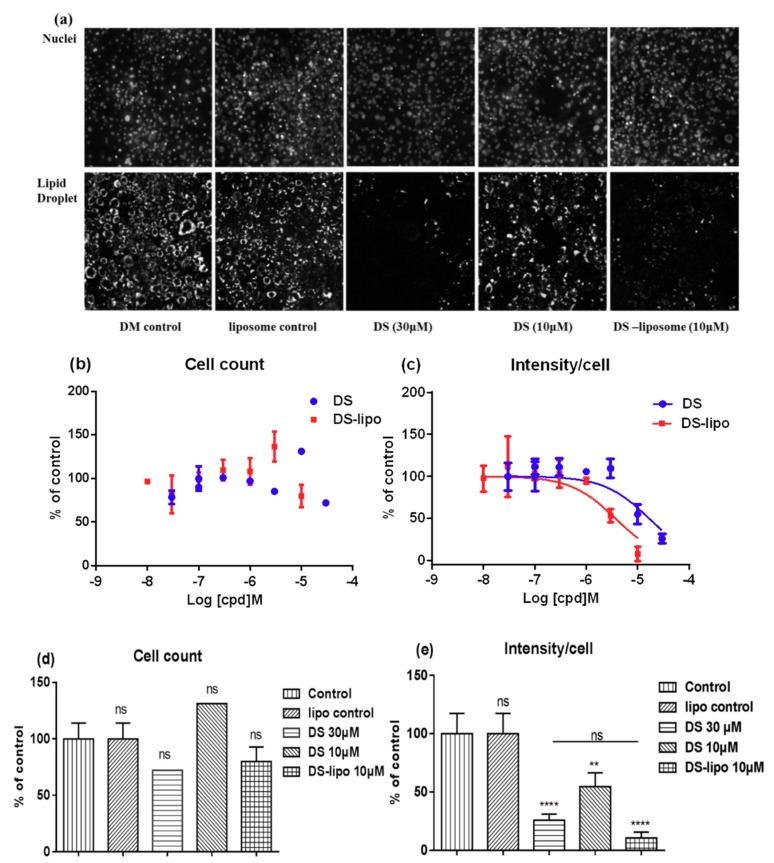
Effect of DS-lipo on lipid droplet formation in 3T3-L1 adipocytes. (**a**) Representative images of cellular lipid droplet and nuclei in 3T3-L1 adipocyte in the presence of DS or DS-lipo. (**b**) Normalized adipocyte cell number treated with various concentrations of DS or DS-lipo. (**c**) Dose response curves of DS and DS-lipo on inhibiting lipid droplet formation. The data was normalized to DM control group and represented mean ±S.D. (*n* = 3). (**d**) and (**e**) The statistical analysis of normalized adipocyte cell number and inhibiting lipid droplet formation of DS and DS-lipo at designated concentration. ns *p* > 0.05, ** *p* < 0.01, **** *p* < 0.001.

Further investigation was to study the suppression–adipogenesis activity of drug-loaded liposomes in 3T3-L1 by the robust phenotype-based high-content screening (HCS) imaging microassay. As shown in Figure 6a, the lipid droplet was remarkably induced in the cytoplasm of 3T3-L1 adipocytes at 10 μM DS-lipo and 30 μM DS.

The effects of DS-lipo and DS on lipid droplet accumulation in 3T3-L1 adipocytes at different concentrations were further studied. As shown in Figure 6c, dose response trends were observed, and less cytoplasmic lipid droplet accumulated with increased concentration treatments. The IC_50_ of inhibiting lipid droplet accumulation effect of DS-lipo and DS compound were 8.68 μM and 31.08 μM, respectively. There were no significant differences between undifferentiated preadipocytes and DS-lipo or DS-treated cells (Figure 6b,e). This meant that neither the DS-lipo nor the DS compound had unapparent cytotoxicity in the whole range of examined concentrations.

Obviously, DS-lipo and DS had a significant capability to inhibit adipocyte differentiation and lipid accumulation in 3T3-L1 preadipocytes when compared to the control group (Figure 6e). Moreover, 10 μM DS-lipo displayed a prominent inhibitory effect on lipid droplet formation. The nanodelivery systems exhibited a definite suppression cytoplasmic lipid droplet accumulation than the original compound, which may contribute to the essential biocompatibility of liposomes.

## 3. Materials and Methods

### 3.1. Chemicals and Reagents

L-α-phosphatidylcholine (Soy) (Avanti Polar Lipids Inc., Alabaster, AL, USA) was used in this study without further purification. DS was purchased from Sichuan Weikeqi Biological Technology Co., Ltd. (Sichuan, China) with purity ≥ 98%, whereas the quantitative analytes were obtained from the National Institutes for Food and Drug Control (Beijing, China). Hepes was purchased from Meryer Chemical Technology Co., Ltd. (Shanghai, China). Methanol and Acetonitrile were purchased from Fisher Scientific. DiR was purchased from Beijing Zhongsheng Ruitai Science & Technology Co., Ltd. (Beijing, China). Milli-Q water (18.2 MΩ·cm) was used in all the experiments. All the vials were washed and sterilized carefully. We obtained 3-(4,5-Dimethyl-2-thiazolyl)-2,5-diphenyl-2H-tetrazolium bromide (MTT) from Merck (Darmstadt, Hesse, Germany).

### 3.2. Animals

Male BALB/c nude mice weighing 20–22 g (aged 6 weeks) were obtained from Vital River Laboratories (Beijing, China). All animals were housed under specific pathogen-free conditions. Water and food were available ad libitum. Ethical approval for all experimental procedures was obtained from the experiment animal administrative committee of the Beijing University of Chinese Medicine.

### 3.3. Liposomes Preparations

The liposomes were prepared using a thin-film hydration method [24]. Briefly, the chloroform solution of the L-α-phosphatidylcholine and cholesterol in a weight ratio of 20:4 and 1 mg of DS at the concentration of 4 mg/mL were added to a 50 mL pyriform flask. Typically, the liposomes were 12 mg/mL total lipid for each sample. The solvent was removed by a vacuum rotary evaporator at 45 °C to form a homogeneous lipid film which was subsequently dried overnight under vacuum for complete removal of the residual solvents. The dry lipid film was then hydrated with 2 mL of PBS buffer (pH 7.4) at 45 °C for 1h by a rotary evaporator (no vacuum) to obtain a crude dispersion of the liposomes. Ultrasound was conducted during the hydration process for 1 min. The sonication amplitude was 100 W and the frequency was 40 KHz. The resulting suspension was then extruded 21 times through a polycarbonate membrane filter of 100 nm with a mini-extruder (Avanti Polar Lipids, Alabaster, AL, USA). The unencapsulated DS was removed from the liposomes using centrifugation at 2000 rpm for 10 min. The centrifuged DS-lipo were stored at 4 °C until further use. For the preparation of liposomes containing fluorescent probe DiR (DiR-liposome), similar procedures were followed.

### 3.4. Liposomes Characterization

#### 3.4.1. Encapsulation Efficiency and Drug Loading

To remove the unloaded free drug from liposomes, a low-speed centrifugation method was developed which cannot affect liposome particle size. Then, the supernatant was diluted into a suitable concentration with methanol and the DS concentration which was retained in reconstituted liposomes was quantified by High Performance Liquid Chromatography (HPLC) analysis as described below.

The concentration of drugs was measured by HPLC analysis simultaneously (Agilent 1100 G1311A pump liquid chromatograph; G1315B Diode-array detector; Diamonsil C_18_ column, 250 mm × 4.6 mm, 5 μm). A constant mobile phase system consisting of Acetonitrile (A) and H_2_O (B) with 0.5% formic acid (80:20, *v/v*) was pumped at a flow rate of 1.0 mL/min with 30 °C column oven temperature, and the column eluents were monitored by signal–wavelength (220 nm). The limit of detection (LOD) and limit of quantification (LOQ) were determined. The values of the slope (b) and the standard deviation (SD) of the y-intercepts (a) of the regression lines were used to calculate the LOD and LOQ [25]:

(1) $$\mathrm{LOD}=\frac{SD_{\left(a\right)}\times 3.3}{b}$$

(2) $$\mathrm{LOQ}=\frac{SD_{\left(a\right)}\times 10}{b}$$

The encapsulation efficiency (EE) and drug loading (DL) were calculated as follows:

(3) $$\mathrm{EE}\left(\%\right)=\frac{\mathrm{Me}}{\mathrm{Mt}}\times 100\%$$

(4) $$\mathrm{DL}\left(\%\right)=\frac{\mathrm{Me}}{\mathrm{Mo}}\times 100\%$$

where Me is the weight of drug loaded in the liposomes, and Mt is the weight of the initial drug added into the formulation. Mo is total weight of lipids.

#### 3.4.2. Laser Light Scattering (LLS)

The size analysis of liposomes was carried out via a commercialized spectrometer from Brookhaven Instruments Corporation (BI-200SM Goniometer, Holtsville, NY, USA) to perform dynamic light scattering (DLS) over a scattering angular range of 20–120°. A vertically polarized, 100 mW solid-state laser (GXC-III, CNI, Changchun, China) operating at 633 nm was used as the light source, and a BI-TurboCo digital correlator (Brookhaven Instruments Corp.) was used to collect and process data. The aqueous solutions of liposomes were filtered through 0.45 μm filters (Sartorius stedim Biotech, Goettingen, Germany) to remove dust, and then monitored by laser light scattering.

#### 3.4.3. Morphology Observations

The formation and morphology of DS-lipo was evaluated by a scanning electron microscope (SEM) in a JSM-IT300 electron microscope, respectively (JEOL, Tokyo, Japan). The samples were placed on aluminum foil substrate and subsequently incubated for 2 h at room temperature (25 °C), prior to microscopy analysis. They were then sputter-coated with gold in a metallizer (JFC-1600, JEOL, Tokyo, Japan) and examined under the SEM operating at an accelerating voltage of 7.0 kV. 

#### 3.4.4. In Vitro Stability Studies of DS-Lipo in Serum

To demonstrate the stability of the liposomes in serum, the liposomes in the presence of 50% fatal bovine serum (FBS) were measured following the procedure described in [26]. Briefly, DS-lipo (phospholipid concentration was about 4 mg/mL) were mixed with an equal volume of FBS and incubated at 37 °C. At 24 h, 100 µL of sample were diluted to 1 mL with a PBS buffer (pH 7.4) for diameter measurements on DLS.

#### 3.4.5. In Vitro Drug Release Studies

The in vitro release of DS from DS-lipo was performed using the dialysis membrane approach. Briefly, 1 mL formulation of free drug solution was put in the cellulose ester dialysis bags (MWCO 8000–14,000 Da, Beijing Biotopped Technology Co. Ltd., Beijing, China). Subsequently, the dialysis membrane bag was totally immersed in 50 mL medium (0.5% (*w/v*) SDS) and a magnetic stirrer was maintained, which was adjusted to a constant release stirring speed of 200 rpm at 37 ± 0.5 °C.

At fixed time intervals, the release medium (0.5 mL) outside the dialysis bag was withdrawn, whereas the isovolumetric fresh medium was replenished to maintain the constant volume. The resulting samples were diluted with methanol, vortexed for 1min, and then filtered through a 0.45 μm filter membrane. The concentration of DS was 0.04 mg/mL equivalent to liposomes formulation in vitro release study.

### 3.5. Fluorescence Live Imaging (FLI)

All in vivo experiments and animal care were approved by the Institutional Animal Care and Use Committee of Beijing University of Chinese Medicine. BALB/c nude mice (6 weeks, 16–20 g) were used for the macrodistrubition study. A recently-introduced carbocyanine lipophilic Near infrared (NIR) fluorescent membrane dye, 1,1-dioctadecyl-3,3,3,3-tetramethylindotricarbocyanine iodide DiR, was used for labeling the cells. DiR is a suitable optical imaging probe with a maximal fluorescence emission in the NIR range [27,28]. DiR-liposome was intravenously injected via the tail vein at a dose of 2 mg/kg for the in vivo and in vitro imaging. In another group, a free DiR-solution (ethanol water (1:5 *v/v*) solution pre-dissolving) was adjusted to the same intensity. Physiological saline was applied as the control. The signal was limited to the abdominal area during the initial period.

In vivo FX-Pro Imaging Systems (Carestream Health, Inc., Toronto, ON, Canada) was employed to record the signal. The excitation and emission filter settings in the IVIS camera system were 760 nm and 790 nm, respectively. Hence, it is a useful labeling technique for nanoparticle tracing experiments. All fluorescence images were acquired with 15 ms exposure time. Anesthesia was maintained with 5% isoflurane in oxygen. FLI was performed at different time points after injection. All the fluorescence intensities were analyzed under the Bruker MISE software. The intensities of the emission spectra were then corrected to account for differences in the excitation absorption derived from the excitation spectra. The average intensity of the fluorescence was calculated for a semiquantitative biodistribution analysis.

### 3.6. Lipid Droplet Accumulation Effect in 3T3-L1 Adipocytes

#### 3.6.1. Lipid Droplet Determination

The differentiation of 3T3-L1 preadipocytes into adipocytes has been widely studied to evaluate the inhibition effect of adipogenesis and lipid accumulation [29]. 3T3-L1 preadipocytes were purchased from American Type Culture Collection (ATCC). The cells were cultured in Dulbecco’s modified Eagles’ medium (DMEM), containing 10% FBS (Gibco, USA), 500 U/mL penicillin, and 500 μg/mL streptomycin at 37 °C in a humidified atmosphere of 5% CO_2_. The differentiation was conducted according to protocol as described in Reference [30]. Plating of cells was designated as Day 1. On Day 4, initiation cell differentiation was conducted with a mixture of 0.5 mM IBMX, 1 mg/mL insulin, 0.25 μM dexamethasone, and 2 mM rosiglitazone. On Day 6, the culture medium was changed to DMEM containing 1 μg/mL insulin for promotion. On Day 8, adipocytes were incubated further in DMEM complete culture medium for 2 days. During the adipocyte differentiation procedure, the adipocytes were incubated with tested DS-lipo and DS compound for another 6 days. 

#### 3.6.2. Lipid Droplet Staining

At the end of cell differentiation (Day 10), the adipocytes were fixed [31]. Then, the cells were replenished with 5 μg/mL Nile red solution for 10 min and stained with final 10 μg/mL Hoechst 33342 solution for another 15 min. After each step, cells were washed three times with phosphate-buffered saline (PBS). Fluorescence intensities of nuclear and lipid droplet were captured by Cellomics Array ScanVTI reader (Thermo Fisher Scientific, Waltham, MA, USA).

### 3.7. Statistical Analyses

All results are expressed as mean ± SD. The statistical analysis was tested by unpaired Student´s *t* test one-way-ANOVA by GraphPad Prism 6.0. A value of *p* < 0.05 was defined as of statistical significance.

## 4. Conclusions

The current study developed a DS nanodelivery system. The prepared DS-lipo was found to be evenly distributed and the particle size was spherical by SEM; the mean particle size of liposomes was about 73.08 nm. The fluorescence live imaging study exhibited hepatic targeting of liposomes in vivo. Fortunately, DS-lipo exhibited a merely prominent inhibitory effect of lipid droplet accumulations, but no influence on cell growth. The inhibition lipid droplet accumulation rate of 10 μM DS-lipo was above 90%. Loading DS into liposomes is an effective way to improve on the poor solubility and limited cellular entry of the drug, thereby providing dosage forms with enhanced therapeutic efficacy.

Traditional herbal medicine is an enormous treasure, which supplies massive natural compounds for drug discovery. The Nobel Peace prize winner Tu Youyou, who found artemisinin, inspired the idea of “Zhouhou Beiji Fang” (《肘后备急方》). The fruit of *Schisandra chinensis*, a traditional Chinese herbal medicine, has been traditionally used as a tonic, sedative, antidiabetic, hepatoprotective, and hypoglycemic agent. Professor Qi Wang, a National Chinese Medical Science Master, screened the optimized formula of Huangjingzanyu capsule based on principle guidelines of liver-based therapy theory, which emphasizes the significance of liver organ treatment. Especially, *Schisandra chinensis*, a main component of the Huangjingzanyu recipe, and deoxyschizandrin is one of the effective active components of the serum.

Liposomes known as the enclosed phospholipid bilayer spherical structure is a versatile vesicular delivery system to carry DS (hydrophobic drug) efficiently into 3T3-L1 preadipocytes. The current study suggests a promising strategy for the treatment of NAFLD and lipid-associated diseases.

## Supplementary Materials

The following Supplementary materials are available on line. Table S1: Levels of factors used in single factor formulation optimization test; Table S2: Experimental results of DS-lipo preparation; Figure S1: Single factor formulation optimization test of factor (A) and factor (B); Figure S2: Single factor formulation optimization test of factor(C).
