# Supplementary material for: Deoxyschizandrin Loaded Liposomes on the Suppression Lipid Accumulation in 3T3-L1 Adipocytes
Source: Molecules. 2018 Aug 27;23(9):2158. doi: 10.3390/molecules23092158 (PMC6225205; doi:10.3390/molecules23092158)
Supplement: Supplementary file 1 [file molecules-23-02158-s001.pdf]

Supplement material

Three mainly effective factors (i.e. the ratio of lipid to chloroform (w/w) (A), the ratio of lipid to DS (w/w) (B) and the lipid concentration (mg/mL) (C) were used in formulation optimization studies (Table 1s) as they were considered to be key ones based on single factor test. The three factors were tested at five different levels choosing encapsulation efficiency of DS as evaluation index.

Table1s Levels of factors used in single factor formulation optimization test

Levels	A(m_{SPC}: m_{Chol})	B(m_{DS}: m_{spc})	C(lipid concentration mg/mL)
1	2:1	4:1	9
2	3:1	8:1	10
3	5:1	10:1	12
4	8:1	15:1	15
5	10:1	20:1	18

Fig. 1s illustrates that the response variable (encapsulation efficiency of DS) ascended at different degree with an increase of ratio of lipid to chloroform (w/w) (A) and the ratio of lipid to DS (w/w) regardless of the factor of the lipid concentration. When the ratio of lipid to chloroform (w/w) (A) and the ratio of lipid to DS (w/w) (B) were 5:1 and 15:1, the encapsulation efficiency of DS was the highest. This phenomenon could be explained that limited space was established between the phospholipid bilayers when the amount of lipoid was fixed.

When the lipid concentration was 12mg/mL, the encapsulation efficiency of DS was the highest, as shown in Fig. 2s.

Thus, the ratio of lipid to chloroform and ratio of lipid to DS were chosen as 5:1 (20: 4) and 20:1. The total lipid concentration was chosen as 12 mg/mL.

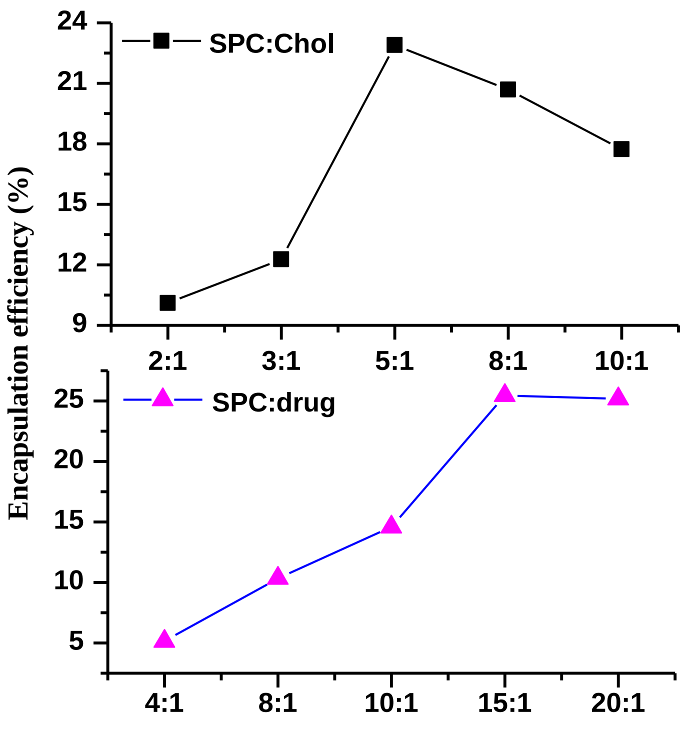

Fig. 1s Single factor formulation optimization test of factor (A) and factor (B)

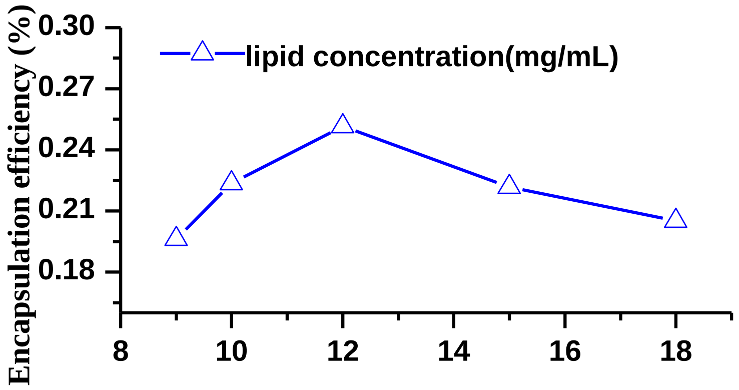

Fig. 2s. Single factor formulation optimization test of factor(C)

The reproducibility of liposome preparation and drug loading had been shown in **Table 2s**. The DS encapsulation efficiency and drug loading were about 24.14±0.19 % and 1.34±0.01 %, respectively.

Table 2s Experimental results of DS-lipo preparation

Code	EE(%)	DL(%)
1	24.29	1.35
2	23.87	1.33
3	24.26	1.35
Mean ± SD	24.14±0.19	1.34±0.01
